# Peripheral blood mononuclear cells from neovascular age-related macular degeneration patients produce higher levels of chemokines CCL2 (MCP-1) and CXCL8 (IL-8)

**DOI:** 10.1186/s12974-017-0820-y

**Published:** 2017-02-23

**Authors:** Judith Lechner, Mei Chen, Ruth E. Hogg, Levente Toth, Giuliana Silvestri, Usha Chakravarthy, Heping Xu

**Affiliations:** 1 Centre for Experimental Medicine, Queen’s University Belfast, Belfast, UK; 2 The Wellcome-Wolfson Institute of Experimental Medicine, Queen’s University Belfast, 97 Lisburn Road, BT9 7BL Belfast, UK

**Keywords:** Age-related macular degeneration, Choroidal neovascularisation, Fibrosis, Cytokines, Chemokines, IL-8, CCL2

## Abstract

**Background:**

Infiltrating immune cells including monocytes/macrophages have been implicated in the pathogenesis of neovascular age-related macular degeneration (nAMD). The aim of this study was to investigate the cytokine and chemokine expression and secretion profile of peripheral blood mononuclear cells (PBMCs) from nAMD patients and the relationship between the cytokine/chemokine expression profile and clinical phenotype of nAMD, including macular fibrosis, macular atrophy or the responsiveness to anti-VEGF therapy.

**Methods:**

One hundred sixty-one nAMD patients and 43 controls were enrolled in this study. nAMD patients were divided into subgroups based on the presence/absence of (1) macular atrophy, (2) macular fibrosis and (3) responsiveness to anti-VEGF therapy; 25–30 ml of peripheral blood were obtained from all participants and 5 ml were used for serum collection, and the remaining were used for PBMC isolation using density gradient centrifugation. Intracellular cytokine expressions by PBMCs following phorbol 12-myristate 13-acetate (PMA) and ionomycin stimulation were examined using flow cytometry. Cytokine productions in lipopolysaccharides (LPS)-or 1% oxygen -treated PBMC were measured using cytometric bead array (CBA) assay. In addition, cytokine and chemokine levels in the serum were also measured by CBA assay.

**Results:**

PBMCs from nAMD patients secreted higher levels of IL-8, CCL2 and VEGF, especially following LPS and 1% oxygen stimulation, than those from controls. 60~80% of IL-8 producing cells were CD11b^+^CD3^−^ monocytes. The percentage of CD11b^+^CD3^−^ IL-8^+^ was significantly increased in nAMD patients compared to controls. PBMCs from nAMD patients without macular fibrosis produced the highest levels of IL-8 and CCL2, whilst PBMCs from nAMD patients with macular atrophy produced highest levels of VEGF. In addition, PBMCs from patients who partially responded to anti-VEGF produced higher levels of IL-8 compared to the cells from complete responders. Interestingly, serum level of CCL2 was not increased in nAMD patients although there was a trend of increased IL-8 in nAMD patients.

**Conclusions:**

PBMCs, in particular monocytes, may contribute to CNV development in nAMD through secreting elevated levels of IL-8, CCL2 and VEGF after they are recruited to the macula. Apart from VEGF, IL-8 and CCL2 may be additional targets for nAMD management.

**Electronic supplementary material:**

The online version of this article (doi:10.1186/s12974-017-0820-y) contains supplementary material, which is available to authorized users.

## Background

Age-related macular degeneration (AMD) is the leading cause of blindness in the elderly in the developed world. Patients with AMD may present with degeneration of retinal pigment epithelium (RPE) and choroid (geographic atrophic, GA) or neovascular membrane and retina and/or RPE detachment (neovascular AMD, nAMD) in the macula. Leakage from neovascular membrane causes macular oedema and visual impairment. Currently, nAMD is treated by intravitreal injections of VEGF inhibitors including ranibizumab (Lucentis, Genentech, San Francisco, CA), bevacizumab (Avastin, Roche, Basel, Switzerland) and more recently aflibercept (Eylea, Regeneron, Tarrytown, NY). Although the therapy can stabilize the disease and even improve vision, not all patients respond to the treatment [1], suggesting that multiple pathways may be involved in the pathogenesis of nAMD.

Epidemiological and genetic studies have shown that ageing, environmental factors (e.g. tobacco smoking, diet, hypertension, cardiovascular disease) [2, 3] as well as gene polymorphisms (e.g. complement-related genes (CFH, C3), Toll-like receptors (TLRs) and chemokine receptors (CX3CR1)) [4–7] increase the risk of AMD. Many of the genes involved in AMD are associated with the immune system, suggesting that dysregulated immune response or inflammation may contribute to AMD aetiology, and this concept is now well appreciated and supported by multiple observations [8]. Inflammatory molecules, including complement components, immunoglobulins and C-reactive protein, have been detected in drusen deposits of AMD patients [9]. Drusen particles or its constituents, such as amyloid beta, can induce inflammasome activation and inflammatory cytokine expression (IL-1β and IL-8) in macrophages and RPE cells [10]. Additionally, inflammatory cytokines (IL-6 and IL-8) have been detected in the aqueous humour of nAMD patients [11].

Inflammation is generally considered as a protective response to infection and tissue injury. When the macula is damaged due to old age and environmental risk factor-mediated oxidative insults, tissue resident immune cells including retinal microglia and choroidal macrophages as well as the complement system may be activated to repair damage and maintain macular function. In addition, circulating immune cells may be recruited to participate in macular inflammation [12]. How the tissue-protective immune response becomes detrimental and induces angiogenesis in nAMD remains unknown. It is believed that the macular microenvironment (high levels of oxidative lipids/proteins and hypoxia) may critically control the level and type of immune response in the ageing eye. We hypothesize that, in addition to macular microenvironment, immune cells in nAMD patients may have a disease-causing phenotype that makes them more pro-inflammatory or angiogenic once recruited to the damaged macula. A recent transcriptome analysis of monocytes has revealed a systemic inflammatory signature in monocytes from nAMD patients [13]. Monocytes from nAMD patients have been shown to express higher levels of chemokine receptor CCR1, CCR2 and CX3CR1 [14] and the activation marker HLA-DR and phosphorylated STAT3 [15]. We have also found that nAMD patients have higher levels of circulating neutrophils [16].

This study was performed to investigate the cytokine/chemokine expression and secretion profile of peripheral mononuclear cells (i.e. monocytes and lymphocytes) from nAMD patients. Considering the diversity of the AMD phenotype as well as the mixed response to anti-VEGF therapy [17], it is feasible that different immune mechanisms may be involved in different types of nAMD. Therefore, we further investigated whether the cytokine profile in nAMD patients was associated with the development of macular fibrosis, macular atrophy or the response to anti-VEGF therapy.

## Methods

### Study participants

The study protocol was approved by the Research Ethics Committee of Queen’s University Belfast and procedures were performed in accordance with the tenets of the Declaration of Helsinki on research into human volunteers. Participants were recruited from the macular disease clinics in Belfast (Belfast Health and Social Care Trust, UK) with written informed consent obtained from each participant. Spouses, relatives or friends who accompanied patients and who were confirmed to be without retinal disease (colour fundus photography (CFP) and optical coherence tomography (OCT)) were recruited as controls. All participants were older than 50 years of age and structured questionnaires were used to ascertain a history of medical conditions, current medication, family history of AMD, smoking habits (current, former smoker, never smoker) and body mass index (BMI). Participants with systemic inflammatory or autoimmune disorders (e.g. patients with active rheumatoid arthritis or active chronic bronchitis) and participants undergoing steroid therapy or chemotherapy were excluded from the study.

The diagnosis of nAMD was by clinical examination and confirmed by multimodal imaging consisting of fundus photography, autofluorescence, OCT, fluorescein angiography and indocyanine green angiography. In this study, most of the participants (158 of 161) were receiving anti-VEGF therapy prior to enrolment. The number of anti-VEGF (ranibizumab, trade name Lucentis, Genentech, San Francisco, CA) injections received by each patient prior to blood collection was ascertained from the medical records.

Participants with nAMD were followed up for 6 months, and we graded colour images and tomographic scans of the recruited participants at their most recent visit prior to closure of the database. Responsiveness to treatment was defined based on the participant achieving a fluid-free macula at any stage during follow-up. In addition, the status of whether a patient was fluid free at the month 3 and month 6 examinations was also recorded. Participants were classified into the following three categories: complete responder: resolution of leakage at any point in time during follow up; partial responder: exhibiting dependence on VEGF inhibitors but a fluid-free macula never achieved; and non-responder: no morphological improvement or worsening. Macular scar identification was based on both colour and OCT characteristics. On colour fundus photographs, macular scar was defined as well-delineated areas of yellowish-white tissue which on OCT corresponded to the presence of linear bands of hyperreflective material that had either obscured or replaced the normal reflectivity and banding of the neurosensory retina and RPE/Bruch's membrane complex. Macular atrophy (MA) was defined as single or multiple areas of hypopigmentation with well-defined borders and visible large choroidal vessels on CFP which corresponded to window defects on angiography and/or to the loss of cellular layers (outer retina, RPE and choriocapillaris) on the accompanying tomograms.

Participant samples were assigned randomly for experimental analysis with some samples being included in more than one analysis. Three experimental analyses were undertaken with the repository of blood samples. Experiment 1 investigated cytokines/chemokines in the serum (133 nAMD and 43 controls samples); experiment 2 investigated secreted cytokines/chemokines by peripheral blood mononuclear cells (PBMCs) (75 nAMD and 28 control samples) and experiment 3 investigated the intracellular cytokine/chemokine expression by PBMCs (28 nAMD and 27 control samples).

### Serum collection

Venous blood (5 ml) was collected in tubes containing serum clot activator and centrifuged at 2000 *g* for 15 min within 3 h of collection. After centrifugation, the serum was aliquoted and stored at −80 °C until analysis.

### PBMC isolation and culture

Whole blood collected (20–25 ml) in tubes containing ethylenediaminetetraacetic acid (EDTA) as anticoagulant between 9:00 am and 12:00 noon was processed within 3 h of collection. PBMCs were isolated by Ficoll-Paque (Histopaque 1077; Sigma-Aldrich, Gillingham, UK) density gradient centrifugation (400 *g* for 30 min at RT with the break turned off) and washed twice with PBS (300 *g* for 10 min at 4 °C). PBMCs were resuspended at 2.5 × 10^6^ cells/ml in RPMI 1640 medium containing 10% FCS and 1% penicillin-streptomycin and cultured in 24-well plates (500 μl/well) and treated immediately with lipopolysaccharides (LPS) (2.5 μg/ml; Sigma-Aldrich) or exposure to hypoxia (1% oxygen) for 16 h. The supernatants for PBMC cultures were collected, centrifuged for 5 min at 300 *g* at 4 °C, and aliquoted and stored at −80 °C until analysis.

### Cytometric bead array

Cytokines were measured in the serum by cytometric bead array (CBA) using CBA Flex Sets (CD121a, CD121b, MCP-1, VEGF, TGFβ1, GM-CSF and IFNα) and CBA Enhanced Sensitivity Flex Sets (IFNγ, IL-2, IL-4, IL-6, IL-8, IL-10, IL12p70, IL-17A and TNFα) (BD Biosciences, Oxford, UK) according to the manufacturer’s instructions. Cytokines in PMBC supernatant were measured using a CBA Human Th1/Th2/Th17 Cytokine Kit (IL-2, IL-4, IL-6, IL-10, TNFα, IFNγ and IL-17A) and CBA Flex Sets (CD121a, CD121b, MCP-1, VEGF, GM-CSF and IL-8) (BD Biosciences) according to the manufacturer’s instructions and as described previously [15]. For cell culture supernatants, the total protein concentration was measured using a Pierce BCA protein assay kit (Thermo Scientific, Loughborough, UK) according to the manufacturer’s instructions. The concentrations of the cytokines were normalized to the total protein concentration (pg/mg total protein). TGF-β1 was measured as a single plex assay and serum samples were activated by incubation with 2.5 N acetic acid/8 M urea for 10 min at RT followed by neutralization with 2.7 N NaOH/1 M HEPES prior to CBA assay according to the manufacturer’s instructions.

### Intracellular cytokine expression of PBMC by flow cytometry

PBMCs were stimulated for 4 h with phorbol 12-myristate 13-acetate (PMA; 100 ng/ml; Sigma-Aldrich) and ionomycin (1 μg/ml; Sigma-Aldrich) in the presence of 1× monensin (BioLegend, UK). After incubation, cells were stained with fluorochrome-labelled antibodies (anti-human CD3-FITC (BD Biosciences), CD3-PE-Cy7, IL-17A-PE, IL-4-APC, IL-6-APC (eBiosciences, San Diego, USA ), IFNγ-APC-Cy7, IL-8-PE, IL-10-Brilliant violet 421 and CD11b-APC-Cy7 (BioLegend, London, UK)).

Briefly, PBMC were washed twice with FACS buffer (300 *g* for 5 min at 4 °C) and resuspended at 10 × 10^6^ cells/ml; 20 μl (2 × 10^5^ cells) were dispensed per FACS tube and incubated with 5 μl Human TruStain FcX (Fc Receptor blocking solution; BioLegend) for 5 min at RT. The cells were then incubated with cell surface antibodies in a total volume of 100 μl FACS buffer for 30 min in the dark at 4 °C. After staining, cells were washed twice with FACS buffer and then fixed and permealized using the Foxp3 Transcription factor Staining Buffer Set (eBiosciences) according to the manufacturer’s instructions. Samples were then incubated with 5 μl Human TruStain FcX (Fc Receptor blocking solution; BioLegend) followed by incubation with intracellular cytokine antibodies in a total volume of 100 μl permealization buffer (eBiosciences) for 40 min in the dark at 4 °C. Cells were washed and acquired on the FACSCantoII flow cytometer (BD Biosciences). Data analysis was performed blindly using FlowJo software version 10.07 for Windows (Tree Star, Oregon, USA).

Gating was performed by first dividing PBMCs into CD11b^+^CD3^−^, CD11b^−^CD3^+^ and CD11b^−^CD3^−^ cells (Fig. 2). Gates for intracellular cytokines IL-4, IL-6, IL-8, IL-10, IL-17A and IFNγ were set on total live cells based on the unstained control, and the same gates were then applied to CD11b^+^CD3^−^, CD11b^−^CD3^+^ and CD11b^−^CD3^−^ cell subsets.

### Statistical analysis

Statistical analysis was performed using the Statistical Package for the Social Sciences, Windows version 21 (SPSS Inc, Armonk, NY). Categorical demographic and clinical data were compared using Pearson’s chi-square test. The distribution of continuous variables was assessed for normality using the Kolmogorov-Smirnov test, and logarithmic transformation was performed if necessary to achieve normal distribution. Normally distributed continuous samples were then compared using the independent samples *t* test or one-way ANOVA. Age was not normally distributed and the difference between controls and nAMD patients was analysed using the Mann-Whitney *U* test.

For the associations that were significant in the univariate analysis, multinomial logistic regression was performed to adjust for age and gender. All variables were also tested for association with family history of AMD, history of cardiovascular disease, history of hypertension, history of diabetes, smoking habits, BMI, taking of cardiovascular medication, vitamins and low-dose aspirin using the independent samples *t* test, one-way ANOVA or Pearson’s correlation. If significant associations were identified, adjustments were made in the multinomial logistic regression analysis. Pearson’s correlation was used to assess the correlation between the number of anti-VEGF injections a patient had received prior to blood collection and the different variables analysed (e.g. cytokine serum levels, secreted cytokine levels and intracellular cytokine levels). Data in figures are presented as mean + standard error of the mean (SEM) calculated from untransformed variables even if the statistical analysis was performed on transformed variables. *P* values <0.05 were considered statistically significant.

## Results

### Clinical evaluation

There was a significant difference in age between controls and nAMD patients in experiment 1 (*P* = 0.001) (Additional file 1: Table S1), but not in experiments 2 (Table 1) and 3 (data not shown). In all three experiments, there were no significant differences regarding gender distribution, family history of AMD, history of cardiovascular disease, history of hypertension, history of diabetes, BMI and smoking habits between controls and nAMD patients. In the serum analysis, there were more nAMD patients taking vitamins compared to controls (Additional file 1: Table S1). Vitamin intake was not significantly increased in experiments 2 and 3 although there was a trend of more participants taking vitamins in the nAMD group compared to controls (Table 1) in both studies. Low-dose aspirin intake was significantly higher in nAMD patients when compared to controls in all three studies (Table 1 and Additional file 1: Table S1).

**Table 1:** Demographic and clinical characteristics of nAMD patients and controls from secreted cytokines analysis (experiment 2)

	All (*n* = 103)	Controls (*n* = 28)	nAMD (*n* = 75)	*P* value nAMD vs control
Age (median (range)), years	77.2 (57–93)	74.5 (59–92)	78.1 (57–93)	0.066^a^
Female sex (number (%))	58 (56)	14 (50)	44 (59)	0.505^b^
Family history of AMD (number (%))	25 (24)	4 (14)	21 (28)	0.202^b^
Cardiovascular disease (number (%))	27 (26)	6 (21)	21 (28)	0.621^b^
Hypertension (number (%))	62 (60)	12 (43)	50 (67)	0.065^b^
Diabetes (number (%))	12 (12)	1 (4)	11 (15)	0.175^b^
Body mass index (mean ± SD)	25.8 (4.0)	25.2 (3.8)	26.1 (4.0)	0.318^c^
Smoking status				0.883^b^
Non-smoker (number (%))	40 (39)	12 (43)	28 (37)	
Former smoker (number (%))	54 (52)	13 (46)	41 (55)	
Current smoker (number (%))	8 (8)	2 (7)	6 (8)	
Taking medication for CVD (number (%))	73 (71)	17 (61)	56 (75)	0.320^b^
Vitamin supplementation (number (%))	21 (20)	2 (7)	19 (25)	0.055^b^
Taking low-dose aspirin (number (%))	33 (32)	3 (11)	30 (40)	*0.008* ^b^

Italics *P* < 0.05

*SD* standard deviation

^a^Mann Whitney *U* test

^b^Pearson’s chi-square test

^c^Independent samples *t* test

Of all patients enrolled, the average duration between the last anti-VEGF treatment and the day of blood collection was 112.5 ± 142.2 days (interquartile range 40.0–118.0 days). No participant had received anti-VEGF treatment within 4 weeks prior to blood collection. The average number of anti-VEGF injections received per nAMD patient prior to blood collection was 15.2 ± 10.3 (interquartile range 8.0–20.0). There was no correlation between the number of anti-VEGF injections received and any of the variables analysed.

### Cytokine levels in the serum in nAMD patients and controls

Of all cytokines measured in the serum IL-8, CD121a, CD121b, CCL2, TGF-β1 and VEGF were detectable whilst the remaining cytokines were below the detection limit (GM-CSF and IFNα <10 pg/ml; IFNγ, IL-2, IL-4, IL-6, IL-10, IL-12p70, IL-17 and TNFα <0.3 pg/ml). There were no significant differences in serum cytokine levels when comparing nAMD patients to controls, although there was a trend of increased IL-8 production in nAMD patients (Table 2).

**Table 2:** Serum cytokine levels in nAMD patients and controls

Variables (pg/ml)	Controls (mean ± SD) *n* = 43	nAMD (mean ± SD) *n* = 133	*P* value nAMD vs controls^a^
IL-8	12.63 ± 9.78	18.17 ± 23.85	0.090
CD121a	1315.88 ± 924.68	1353.27 ± 988.96	0.829
CD121b	2601.10 ± 1601.77	2477.91 ± 1313.55	0.652
CCL2	177.00 ± 253.49	138.57 ± 77.25	0.380
TGF-β1	7930.12 ± 4467.25	8200.52 ± 3613.87	0.757
VEGF	68.18 ± 56.49	92.67 ± 89.07	0.117

*SD* standard deviation

^a^Independent samples *t* test

### Cytokine production by PBMCs from nAMD and controls

**Fig. 1:**
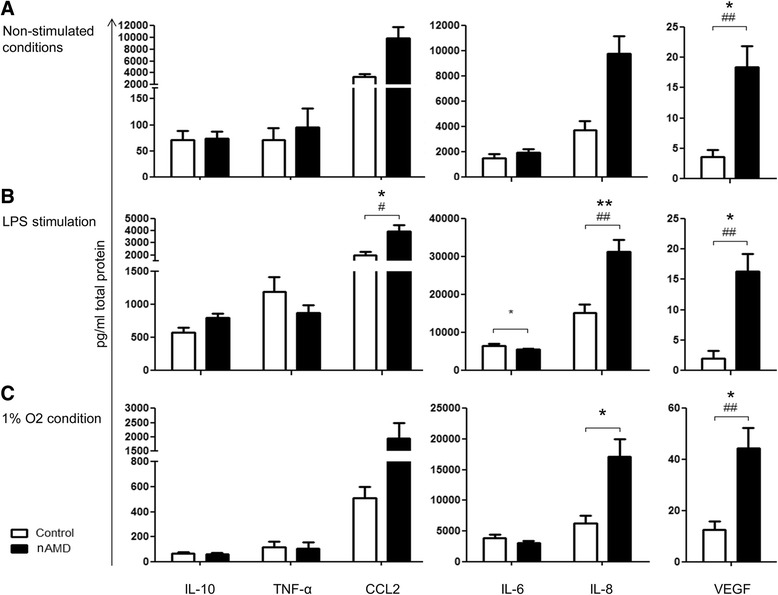
Cytokine secretion by PBMC. IL-10, TNFα, CCL2, IL-6, IL-8 and VEGF secretion in the supernatant of PBMCs from controls and nAMD patients under non-stimulated conditions (**a**), following LPS treatment (**b**) and under hypoxic conditions (**c**). Controls *n* = 28, nAMD *n* = 75; mean + SEM; #*P* < 0.05, ##*P* < 0.01 in univariate analysis using independent samples *t* test; *P < 0.05, **P < 0.01 in multivariate analysis using multinomial logistic regression

Of all cytokines measured in the supernatants of PBMC cultures, IL-10, TNFα, CCL2, IL-6, IL-8 and VEGF were detectable whilst the remaining cytokines were below the detection limit (CD121a and CD121b <40 pg/ml; GM-CSF <10 pg/ml; IL-2, IL-4, IFNγ and IL-17A <20 pg/ml). Under non-stimulated conditions, PBMCs from nAMD patients appeared to secrete higher levels of CCL2 and IL-8 although statistically insignificant when compared to controls (Fig. 1a). VEGF was significantly increased in PBMC supernatants from nAMD compared to controls in both univariate and multivariate (adjusting for age and gender) analyses (Fig. 1a).

Following LPS stimulation the production of IL-10, TNFα, IL-6, and IL-8 was significantly increased (Fig. 1b vs Fig. 1a). There was a significant increase in CCL2, IL-8 and VEGF in PBMC supernatants from nAMD patients compared to controls in the univariate and multivariate analysis (corrected for age and gender, as well as low-dose aspirin intake) (Fig. 1b). Interestingly, IL-6 production was significantly reduced in PBMC supernatants from nAMD patients compared to controls following LPS stimulation.

The 1% oxygen treatment significantly increased the production of VEGF but not other cytokines in PBMC from both control and nAMD patients (Fig. 1c vs Fig. 1a). Under 1% oxygen condition, PBMCs from nAMD patients secreted more IL-8 and VEGF compared to those from controls in both univariate and multivariate analysis (Fig. 1c).

Taken together, our results show that PBMCs from nAMD patients produce more angiogenic growth factor VEGF and chemokines MCP-1 (CCL2) and IL-8 (CXCL8) than those from healthy controls, particularly under inflammatory conditions (e.g. LPS stimulation).

### Intracellular cytokine production by PBMC

To further understand which subsets of PBMC produced higher levels of IL-8 in nAMD patients, we examined intracellular cytokine expression using flow cytometry. Cell surface antigens CD11b and CD3 were used to differentiate CD11b^+^CD3^−^ (predominately monocytes), CD11b^−^CD3^+^ (predominately T cells) and CD11b^−^CD3^−^ cells (predominately B cells) (Additional file 2: Figure S1A). Naïve PBMCs consisted of ~20% CD11b^+^CD3^−^ cells, 50–60% CD11b^−^CD3^+^ and 20–30% CD11b^−^CD3^−^ cells. After simulation with PMA/ionomycin for 16 h, there was a slight shift towards more CD11b^−^CD3^−^ cells and less CD11b^+^CD3^−^ cells in both nAMD patients and controls (Additional file 2: Figure S1B). This shift towards a lower percentage of monocytes following PMA/ionomycin stimulation has been reported previously (~22% CD14^+^ cells before stimulation vs 12% CD14^+^ cells after stimulation) [18].

**Fig. 2:**
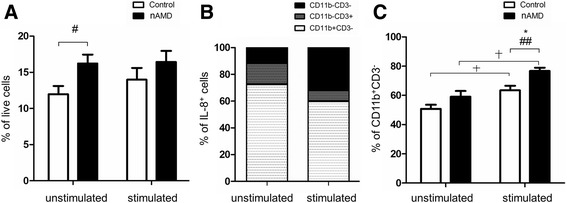
IL-8 producing PBMCs under non-stimulated conditions and after stimulation with PMA/ionomycin. Percentage of total IL-8 producing PBMCs from controls and nAMD patients (**a**) and composition of IL-8 producing cells from all samples (**b**). Percentage of CD11b^+^CD3^-^ cells that were positive for intracellular IL-8 from controls and nAMD patients (**c**). Controls *n* = 27, nAMD = 28; mean + SEM; #*P* < 0.05, ##*P* < 0.01 in univariate analysis suing independent samples *t* test; *P < 0.05 in multivariate analysis using multinomial logistic regression

In naïve non-stimulated PBMCs, the percentage of IL-8-producing cells was significantly increased in nAMD patients compared to controls in univariate (*P* = 0.017) but not multivariate analysis (adjusting for age, gender, cardiovascular drug and low-dose aspirin intake, *P* = 0.089, Fig. 2a). After stimulation with PMA/ionomycin, the percentage of IL-8 producing cells remained higher in nAMD patients compared to controls; however, the difference was not statistically significant. Further analysis showed that 60–80% of IL-8-producing cells were CD11b^+^CD3^−^ monocytes (Fig. 2b), suggesting that monocytes are the major sources of IL-8 production in PBMC cultures. Following PMA/ionomycin treatment, significantly higher populations of CD11b^+^CD3^−^ cells produced IL-8 in both controls and nAMD patients (Fig. 2c). The percentage of IL-8-producing CD11b^+^CD3^−^ cells was significantly higher in nAMD patients compared to controls after PMA/ionomycin stimulation in both univariate and multivariate analysis (Fig. 2c). In addition, the percentage of IL-8-producing CD11b^−^CD3^+^ T cells was higher in nAMD patients (5.0 ± 4.3%) compared to controls (2.9 ± 2.5%) in the univariate (*P* = 0.038 ), but not multivariate analysis (*P* = 0.117, adjusting for age, gender and diabetes).

Our results suggest that increased IL-8 production by PBMCs (Fig. 2) in nAMD patients may attribute predominately to CD11b^+^CD3^−^ monocytes.

**Fig. 3:**
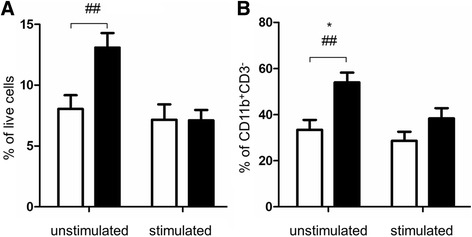
IL-6 producing PBMCs under non-stimulated conditions and after stimulation with PMA/ionomycin. Percentage of total (**a**) and CD11b^+^CD3^-^ (**b**) IL-6 producing PBMCs from controls and nAMD patients. Controls *n* = 27, nAMD = 28; mean + SEM; ##*P* < 0.01 in univariate analysis using independent samples *t* test; *P < 0.05 in multivariate analysis using multinomial logistic regression

Although there was no significant difference in IL-6 levels in PBMC supernatants from non-stimulated cells between nAMD and controls (Fig. 1a), the population of IL-6 producing cells, in particular CD11b^+^CD3^−^IL-6^+^ cells, was significantly increased in nAMD patients under non-stimulatory conditions (Fig. 3).

There were no significant differences between nAMD and controls in IL-4, IL-10, IFNγ or IL-17A producing cells under naïve or PMA/ionomycin treatment conditions (Additional file 2: Figure S2).

### The production of CCL2 and IL-8 by PBMCs and macular fibrosis

**Fig. 4:**
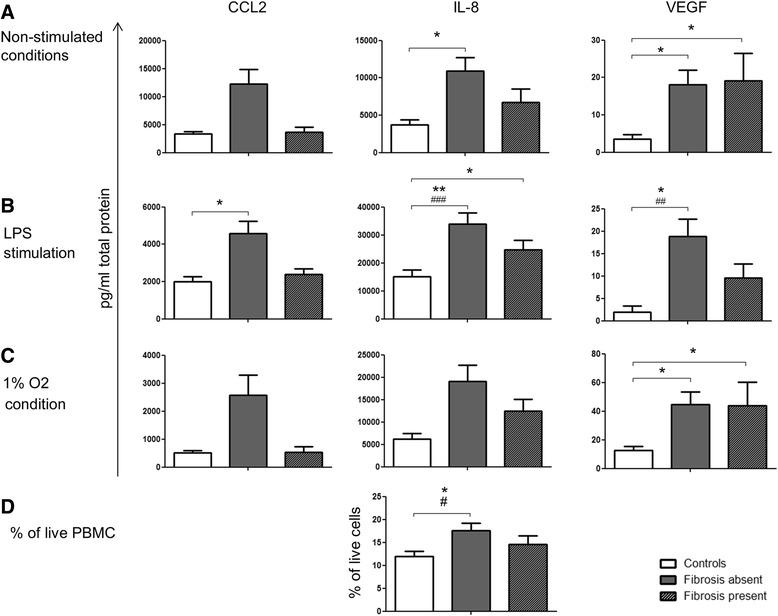
Cytokine production and macular fibrosis. CCL2, IL-8 and VEGF levels in PBMC supernatants under non-stimulated condition (**a**), following LPS treatment (**b**) and under hypoxic condition (**c**) as well as percentage of total IL-8 producing PBMCs under non-stimulated conditions (**d**) from healthy controls and nAMD patients without and with fibrosis. Supernatants: controls, *n* = 28, fibrosis (−), *n* = 54, fibrosis (+), *n* = 21; PBMC: controls, *n* = 27, fibrosis (−), *n* = 15, fibrosis (+), *n* = 13; mean + SEM; #*P* < 0.05, ##*P* < 0.01, ###*P* < 0.001 in univariate analysis using one-way ANOVA; *P < 0.05, **P < 0.01 in multivariate analysis using multinomial logistic regression

To understand the link between CCL2/IL-8 and macular fibrosis, nAMD patients were sub-grouped into fibrosis absent (−) and present (+). PBMCs from fibrosis (−) patients secreted the highest levels of CCL2 and IL-8 compared to cells from other groups (Fig. 4), particularly after LPS stimulation (Fig. 4b). The higher level of IL-8 by PBMCs from fibrosis (−) patients was further confirmed by intracellular cytokine staining (Fig. 4d). Secreted VEGF levels were similar in both patient groups and were elevated when compared to controls (Fig. 4).

No differences were observed for IL-10, TNFα and IL-6 levels produced by PBMCs between different groups of nAMD patients and controls.

### Cytokine production by PBMC and MA

**Fig. 5:**
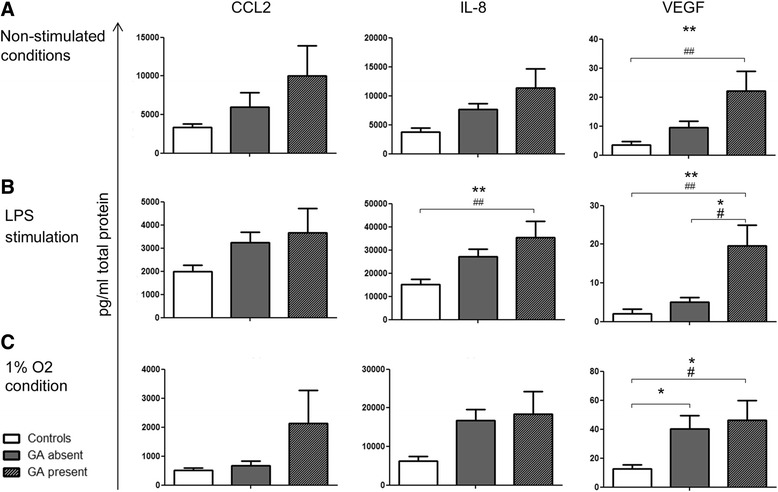
Cytokine production and macular atrophy. CCL2, IL-8 and VEGF levels in PBMC supernatants from healthy controls and nAMD patients without and with macular atrophy (MA) under non-stimulated condition (**a**), following LPS treatment (**b**) and under hypoxic condition (**c**). Controls *n* = 28, MA (−) *n* = 17, MA (+) *n* = 35; mean + SEM; #*P* < 0.05, ##*P* < 0.01 in univariate analysis using one-way ANOVA. *P < 0.05, **P < 0.01 in multivariate analysis using multinomial logistic regression

Amongst the 75 nAMD patients whose cytokine production by PBMCs was studied, information regarding MA at the time of sample collection was available from 52 patients, of which 35 had MA and 17 did not have MA. The level of CCL2 in PBMC supernatants did not appear to be related to the presence or absence of MA (Fig. 5). IL-8 was significantly increased in nAMD patients with MA compared to controls following LPS stimulation. Interestingly, higher levels of VEGF production by PBMC, particularly after LPS stimulation, appeared to be related to MA development (Fig. 5b). The number of anti-VEGF injections appeared to be higher in the MA present group compared to the MA absent group although statistically insignificant (*P* = 0.056).

### Cytokine production by PBMCs and response to anti-VEGF therapy

**Fig. 6:**
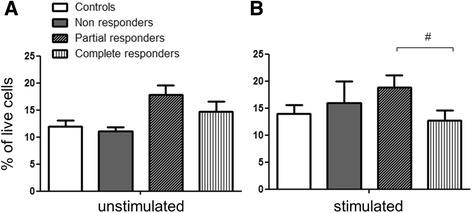
IL-8 producing PBMC and response to anti-VEGF therapy. Percentage of IL-8 producing PBMCs of controls and nAMD patients not, partially and completely responding to anti-VEGF therapy  under non-stimulated condition (**a**), and following PMA/ionomycin stimulation (**b**). Controls:*n* = 27, non-responders: *n* = 2, partial responders: *n* = 16, complete responders: *n* = 10; mean + SEM; #*P* < 0.05, in univariate analysis using independent samples *t* test

Of the 75 nAMD patients whose PBMCs were studied, 41 responded partially, 32 responded completely and 2 did not respond to anti-VEGF (ranibizumab/Lucentis) therapy. No significant differences were found when comparing secreted cytokine levels in the PBMC supernatants from patients partially and completely responding to anti-VEGF therapy. Following PMA/ionomycin stimulation, the population of IL-8-producing cells was increased in partial responders compared to complete responders in the univariate but not multivariate analysis (Fig. 6). The non-responder group was not included in the statistical analysis due to low sample size (*n* = 2).

## Discussion

Inflammation is known to play a critical role in the development of choroidal neovascularisation in AMD although the underlying mechanism is not fully understood [8, 19, 20]. Inflammatory cells may migrate from surrounding tissues including the neuronal retina (i.e. microglia) and choroid to the macula or be recruited from circulating immune cells [12]. In this study, we provide evidence that PBMCs, in particular monocytes from nAMD patients, produce increased levels of IL-8, CCL2 and VEGF, especially under inflammatory conditions. Our results suggest that PBMCs in nAMD patients may contribute to the development of macular angiogenic lesions through producing excessive amounts of IL-8, CCL2 and VEGF.

IL-8 (CXCL8), an important inflammatory cytokine and potent angiogenic factor, has previously been linked to the pathogenesis of AMD. Single-nucleotide polymorphisms (SNPs) in the IL-8 regulatory and coding regions have been associated with increased risk of AMD [21–23] and certain SNP genotypes have been linked to increased IL-8 serum levels [24]. Exactly how IL-8 contributes to AMD pathology is not known. Previous studies have reported elevated levels of IL-6, IL-8 and CCL2 in the aqueous humour from nAMD patients [25], and the levels correlated with choroidal neovascularisation (CNV) lesion size [26]. Although retinal cells, including microglia, vascular endothelial cells [27] and RPE cells [28], can produce IL-8, our results suggest that infiltrating immune cells, especially CD11b^+^CD3^−^ monocytes may contribute to increased intraocular IL-8 production in nAMD.

IL-8 is known to be involved in both physiological and pathological angiogenesis through its receptors CXCR1 and CXCR2 [29]. The pro-angiogenic effect of IL-8 on endothelial cells is mediated partially through the induction of VEGFR2 and VEGF-A expression [30] although a VEGF-independent pathway has also been reported [31]. IL-8 production has been associated with resistance to anti-VEGF therapy in tumours, further supporting a VEGF-independent pro-angiogenic mechanism for IL-8 [32]. In this study, we observed increased levels of IL-8 by PBMCs from nAMD patients who are partially resistant to anti-VEGF therapy compared to PBMCs from complete responders (although the difference was not statistically significant, possibly due to insufficient patient numbers). The results suggest that apart from VEGF, IL-8 may be another important pro-angiogenic factor in nAMD. Further studies using larger patient samples are necessary to confirm the role of IL-8 in nAMD and the response to anti-VEGF therapy.

Previously, we reported increased levels of neutrophils in nAMD patients [16]. IL-8 is a strong chemotactic factor for neutrophils [33]. Although how neutrophils are involved in nAMD is not known, the high levels of IL-8 by PBMC may contribute to increased levels of circulating neutrophils in nAMD.

CCL2 plays a crucial role in monocyte and lymphocyte trafficking under inflammatory conditions [34]. Compelling evidence suggests that the CCL2/CCR2 pathway may play a role in AMD pathogenesis. The aqueous humour of nAMD patients contains high levels of CCL2 [35], and CCL2 expression was detected in atrophic lesions in AMD patients [36]. Monocytes from nAMD patients were shown to express higher levels of CCR1 and CCR2 [14]. Importantly, experimental studies have shown that CCL2 can promote CNV formation through enhanced recruitment of myeloid cells [37]. In this study, we found that PBMCs from nAMD patients produced higher levels of CCL2, although there was no difference in the serum CCL2 concentration between nAMD patients and controls (which is in line with a previous report [14]). Our results indicate that CCL2 production by infiltrating immune cells might be related to macular lesion development in nAMD.

In a previous study, we reported increased STAT3 activation in circulating monocytes from nAMD patients [15], which may contribute to increased VEGF production by PBMC observed in the current study. IL-6 is one of the major cytokines that can active the JAK1/STAT3 pathway. Interestingly, in this study, we found an increased percentage of IL-6 producing CD11b^+^CD3^−^ monocytes in nAMD patients compared to that in controls. A previous study has reported an association between circulating levels of IL-6 (~6 pg/ml) and AMD progression [38]; however, in our study, the serum levels of IL-6 were below the detection limit, i.e. 0.3 pg/ml in both patients and controls. The role of IL-6 in nAMD warrants further investigation.

The development of macular fibrosis results in irreversible vision loss in nAMD [39], although the underlying mechanisms remain ill-defined and there is no treatment modality for it. Even following the introduction of anti-VEGF therapies, which have improved visual outcomes markedly, fibrosis remains an integral component of the macular lesion and has been shown to be associated with the poorer outcomes compared to eyes without fibrosis [40–42]. In the present study, we observed increased levels of IL-8 and CCL2 in PBMCs from nAMD patients without fibrosis suggesting that IL-8 and CCL2 from PBMCs might reduce the risk. Interestingly, IL-8 and CCL2 have previously been shown to have pro-fibrotic effects in various tissues including the lung [43, 44], kidney [45, 46], liver [47] and skin [48, 49]. The tissue-specific role of CCL2 and IL-8 in fibrosis has not been reported before. Therefore, it is possible that those chemokines may have anti-fibrotic effects in the retinal microenvironment. Other chemokines such as CXCL10 have previously been shown to mediate tissue-specific pro- (e.g. in the liver) [50] as well as anti-fibrotic effects (e.g. in the lung) [51]. Further studies are necessary to investigate the role of CCL2 and IL-8 in fibrosis in nAMD.

Macular atrophy (MA) is due to the degeneration of RPE and overlying photoreceptors although the underlying mechanism remains poorly defined. MA and nAMD co-exist in many patients, particularly following long-term anti-VEGF therapy [1]. In the present study, 67% of patients exhibited MA during their follow-up. PBMC from the patients who had MA produced increased levels of VEGF. Previous studies have shown that enhanced MA progression in nAMD patients is associated with anti-VEGF injections [52]. In the present study, a numerically greater proportion of patients with MA had received more anti-VEGF injections than those without but this did not reach statistical significance (*P* = 0.056). The reasons for the higher prevalence of MA in eyes undergoing anti-VEGF treatment is not entirely explicable by a higher rate of exposure to VEGF but may also reflect destruction of the macular tissues by a more active CNV lesion and hence the higher number of treatments required in this group [41].

The strengths of the present study include independent grading of AMD, fibrosis, MA and anti-VEGF responsiveness and systematic and extensive exploration of cytokine production and secretion by PBMCs in nAMD as well as in patients with fibrosis, MA and partially or completely responding to anti-VEGF treatment.

One of the limitations of this study is that patients were recruited to the study at different stages of nAMD, and therefore, some patients classified as not having macular fibrosis or atrophy may do so over time. Secondly, 98% of patients were on anti-VEGF treatment at the time of enrolment which might have an effect on circulating immune cells although we did not find any correlation between variables analysed and the number of anti-VEGF injections received. Despite our efforts to recruit age-matched controls, there was a significant difference in age between nAMD patients and controls in one of our experimental analyses (serum analysis, experiment 1). Finally, some of the analyses were done with small patient numbers (e.g. patients without MA *n* = 17, partial responders *n* = 16, complete responders *n* = 10) and larger confirmatory studies are necessary to verify our results.

## Conclusion

In summary, our study shows that PBMCs, especially monocytes, appear to be pre-conditioned to an inflammatory and angiogenic phenotype in nAMD patients. They may contribute to dysregulated retinal inflammation [20] and CNV formation after recruitment to the macula by secreting elevated levels of IL-8, CCL2 and VEGF. Our research also uncovered a link between PBMC released IL-8 and CCL2 and macular fibrosis in nAMD. Further studies are necessary to understand whether IL-8 and CCL2 might be targeted for nAMD management.

## Additional files

Additional file 1: Table S1. Demographic and clinical characteristics of nAMD patients and controls from serum study (study 1). (PDF 112 kb)
Additional file 2: Figure S1. Flow cytometry analysis of PBMCs. PBMCs were first divided into CD11b^+^CD3^−^, CD11b^−^CD3^+^ and CD11b^−^CD3^−^ cells (A) and the average percentage of all samples (*n* = 55) was analysed before and after stimulation with PMA/ionomycin (B). **Figure S2.** Percentage of total IL-4 and IL-10 producing PBMCs and percentage of CD11b^−^CD3^+^ IL-17A and IFNγ producing PBMCs (almost all of IL-17A and IFNγ producing PBMCs were CD11b^−^CD3^+^) from controls and nAMD patients under non-stimulated culture conditions and after stimulation with PMA/ionomycin. Controls *n* = 27, nAMD = 28; mean + SEM are shown. (PDF 413 kb)

## Abbreviations

AMD: Age-related macular degeneration
BMI: Body mass index
CFP: Colour fundus photography
CNV: Choroidal neovascularisation
MA: Macular atrophy
nAMD: Neovascular AMD
OCT: Optical coherence tomography
PBMC: Peripheral blood mononuclear cell
RPE: Retinal pigment epithelium
VEGF: Vascular endothelial growth factor
