# Supplementary material for: Peripheral blood mononuclear cells from neovascular age-related macular degeneration patients produce higher levels of chemokines CCL2 (MCP-1) and CXCL8 (IL-8)
Source: J Neuroinflammation. 2017 Feb 23;14:42. doi: 10.1186/s12974-017-0820-y (PMC5324243; doi:10.1186/s12974-017-0820-y)
Supplement: Additional file 1: Table S1. — Demographic and clinical characteristics of nAMD patients and controls from serum study (study 1). (PDF 112 kb) [file 12974_2017_820_MOESM1_ESM.pdf]

Table S1: Demographic and clinical characteristics of nAMD patients and controls from serum study (study 1)

	**All (n = 176)**	**Controls (n = 43)**	**nAMD (n = 133)**	***P* value nAMD vs Control**
Age (median (range)), years	79.6 (53-93)	74.0 (58-92)	80.4 (53-93)	**0.001**[1]
Female sex (number (%))	86 (49)	19 (44)	67 (50)	0.480[2]
Have history of AMD (number (%))	39 (22)	6 (14)	33 (25)	0.153[2]
Have history of cardiovascular disease (number (%))	48 (27)	9 (21)	39 (29)	0.317[2]
Have history of hypertension (number (%))	106 (60)	23 (53)	83 (62)	0.377[2]
Have history of diabetes (number (%))	21 (12)	2 (5)	19 (14)	0.098[2]
Body Mass Index (mean ± SD)	26.0 (4.6)	26.1 (5.1)	26.0 (4.4)	0.912[3]
Smoking status				0.731[2]
Non-smoker (number (%))	76 (43)	20 (47)	56 (42)	
Former smoker (number (%))	87 (50)	20 (47)	67 (50)	
Current smoker (number (%))	12 (7)	2 (5)	10 (8)	
Taking cardiovascular medication (number (%))	129 (73)	28 (65)	101 (76)	0.234[2]
Taking vitamins (number (%))	39 (22)	3 (7)	36 (27)	**0.007**[2]
Taking low-dose aspirin (number (%))	57 (32)	5 (12)	52 (39)	**0.001**[2]

[1] Mann Whitney U test
[2] Pearson's chi-square test
[3] Independent samples t-test
SD: Standard deviation; **Bold** *P*<0.05
