# Supplementary material for: Peripheral blood mononuclear cells from neovascular age-related macular degeneration patients produce higher levels of chemokines CCL2 (MCP-1) and CXCL8 (IL-8)
Source: J Neuroinflammation. 2017 Feb 23;14:42. doi: 10.1186/s12974-017-0820-y (PMC5324243; doi:10.1186/s12974-017-0820-y)
Supplement: Additional file 2: Figure S1. — Flow cytometry analysis of PBMCs. PBMCs were first divided into CD11b+CD3−, CD11b−CD3+ and CD11b−CD3− cells (A) and the average percentage of all samples (n = 55) was analysed before and after stimulation with PMA/ionomycin (B). Figure S2. Percentage of total IL-4 and IL-10 producing PBMCs and percentage of CD11b−CD3+ IL-17A and IFNγ producing PBMCs (almost all of IL-17A and IFNγ producing PBMCs were CD11b−CD3+) from controls and nAMD patients under non-stimulated culture conditions and after stimulation with PMA/ionomycin. Controls n = 27, nAMD = 28; mean + SEM are shown. (PDF 413 kb) [file 12974_2017_820_MOESM2_ESM.pdf]

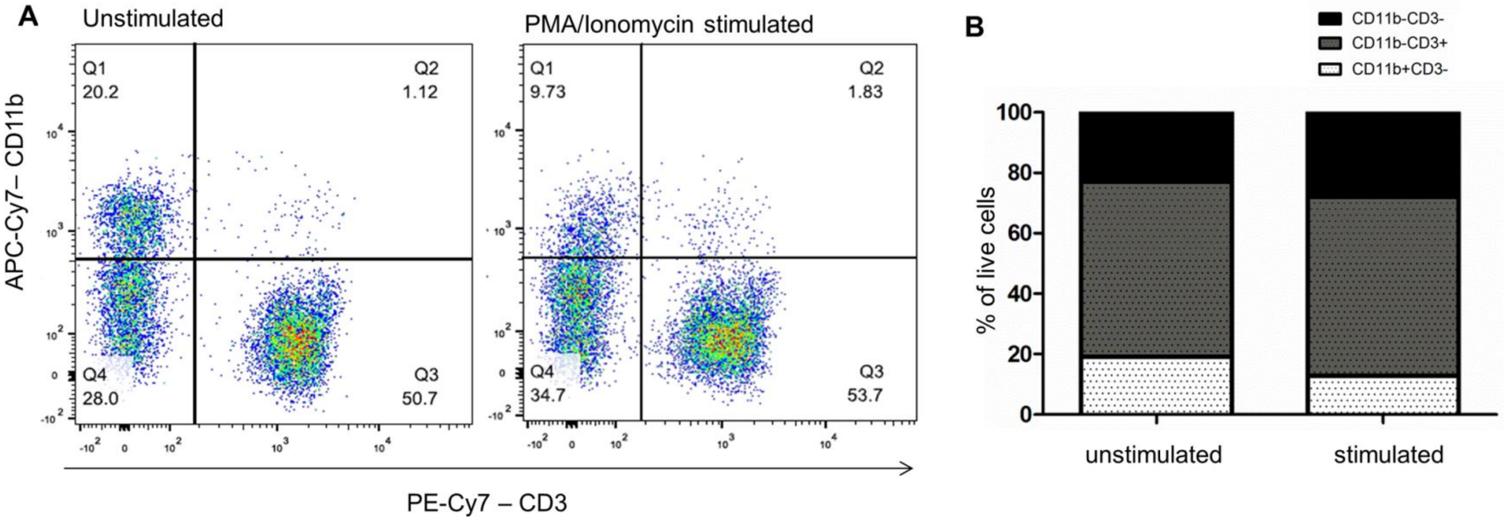

Figure S1: Flow cytometry analysis of PBMCs. PBMCs were first divided into CD11b$^+$CD3$^-$, CD11b$^-$CD3$^+$ and CD11b$^-$CD3$^-$ cells (A) and the average percentage of all samples (n = 55) was analysed before and after stimulation with PMA/ionomycin (B).

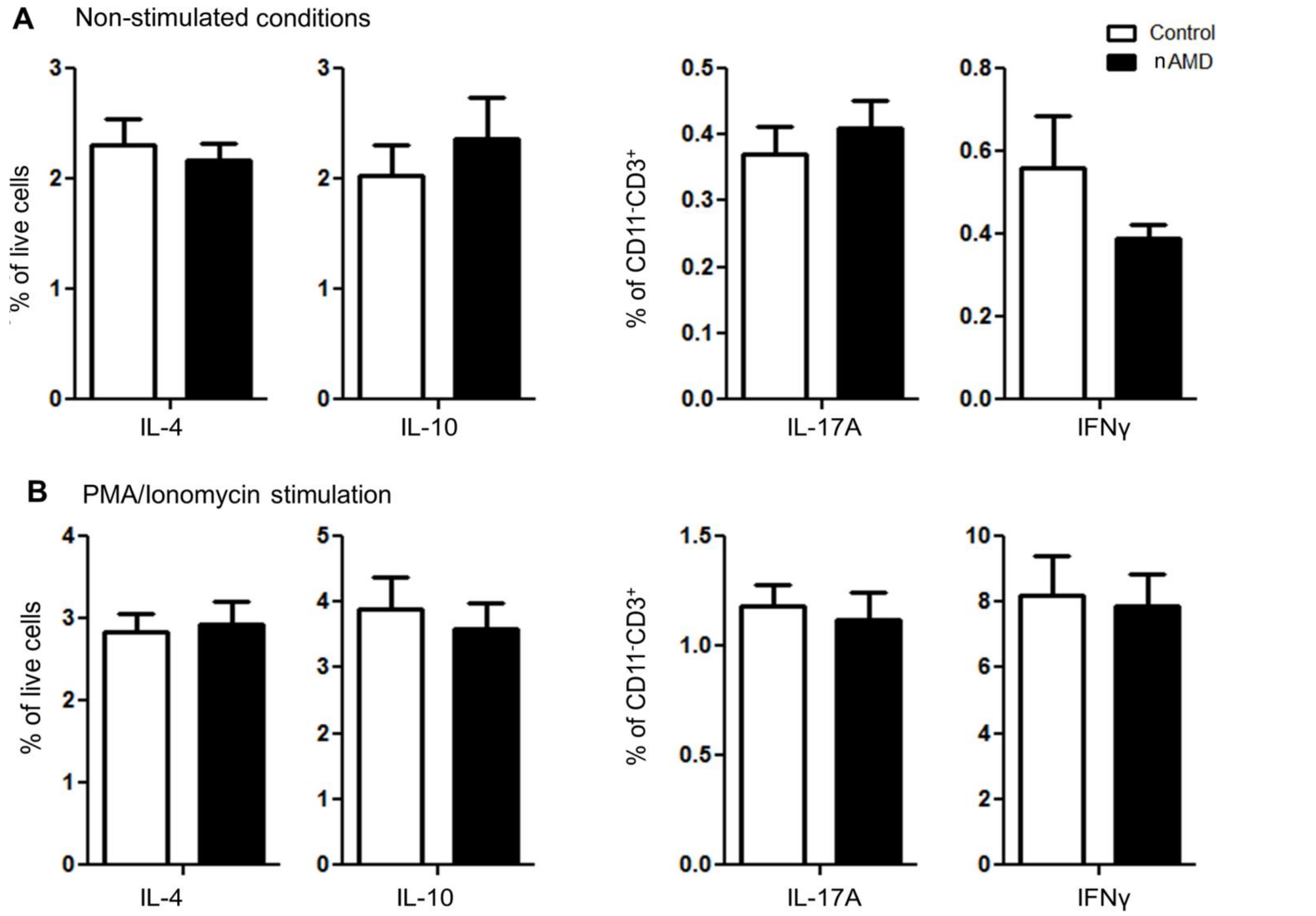

Figure S2: Percentage of total IL-4 and IL-10 producing PBMCs and percentage of CD11b$^-$CD3$^+$ IL-17A and IFNγ producing PBMCs (almost all of IL-17A and IFNγ producing PBMCs were CD11b$^-$CD3$^+$) from controls and nAMD patients under non-stimulated culture conditions and after stimulation with PMA/ionomycin. Controls n = 27, nAMD = 28; mean + SEM are shown.
